# Placental pathologic lesions associated with stroke in term neonates

**DOI:** 10.3389/fendo.2022.920680

**Published:** 2022-09-08

**Authors:** Rachel L. Leon, Vinay Kalvacherla, Michelle Machie Andrews, Jennifer M. Thomas, Imran N. Mir, Lina F. Chalak

**Affiliations:** ^1^ Department of Pediatrics, University of Texas Southwestern Medical Center, Dallas, TX, USA; ^2^ University of Texas Southwestern Medical School, Dallas, TX, USA

**Keywords:** perinatal stroke, placenta, chorioamnionitis, villitis, perinatal asphyxia, vascular malperfusion, neonatal brain, neonatal seizure

## Abstract

**Objective:**

To determine the birth prevalence of perinatal stroke in term born infants at our high-volume delivery center and assess the frequency of both gross and histologic placental pathologies associated with perinatal stroke using the Amsterdam Placental Workshop Group Consensus Statement guidelines and definitions.

**Study Design:**

A single-center retrospective cohort study spanning 2010-2020.

**Results:**

There were 129,759 live births at Parkland Hospital during the study period and a total of 18 term born infants leading to a birth prevalence of 1 in 6,829 infants. Perinatal risk factors were found in all but one patient, and 74% presented with seizures. Pathologic placental examination was available in 56% of the cohort and only one patient had normal placental examination. Acute histologic chorioamnionitis was described in five placentas (50%) and an additional two had isolated umbilical and/or chorionic plate vasculitis with or without funisitis compared to a rate of 28% with acute inflammation in a Control group. Chronic inflammation in the form of villitis of unknown etiology was described in three of the acutely inflamed placentas and was high-grade in each of those while none of the placentas from our Control group showed evidence of any chronic lesion.

**Conclusion:**

Both acute and chronic placental inflammation are common in perinatal stroke; placental examination should be considered an essential component to the diagnostic workup.

## Introduction

Perinatal stroke is defined as cerebrovascular injury occurring between 20 weeks gestational age until the 28^th^ postnatal day. Perinatal stroke affects an estimated 1 in 2,300 to 1 in 5,000 term born infants (1–3). In broad terms, perinatal stroke comprises several entities including arterial ischemic stroke (AIS), cerebral sinovenous thrombosis (CSVT), and intracranial hemorrhage (ICH). Perinatal AIS is the most common form of stroke in term neonates and is a leading cause of hemiplegia in pediatric patients (4, 5). Perinatal AIS is characterized by imaging findings of parenchymal infarct in a specific arterial territory, and outcomes vary widely depending on timing of injury, lesion size, and location (6–8). Perinatal CSVT is less common entity most frequently affecting the sagittal and/or lateral sinus (9–11).

Numerous risk factors have been associated with perinatal stroke (12–20), which can be divided into maternal, fetal, and neonatal complications. Suspected maternal risk factors include preeclampsia and prothrombotic disorders, while fetal risk factors include intrauterine growth restriction, prolonged rupture of membranes, and congenital heart disease. Neonatal factors associated with perinatal stroke involve birth asphyxia, infection, and hematologic disorders. Despite numerous studies attempting to define *risk factors* for perinatal stroke, the etiology of the injury remains unclear in most cases. Some experts hypothesize that for AIS, a thromboembolus may originate from the placenta, enter fetal circulation, and cross the patent foramen ovale to result in cerebral artery obstruction (21), but no studies to date have confirmed this mechanism (22, 23). As much as 30% of neonatal AIS is multifocal, even in the absence of congenital cardiac disease (4), and recurrence postnatally is exceedingly rare, supporting this hypothesis of a placental origin of thromboembolism. The connection of the placenta to CSVT and ICH is less clear, but may be associated with chronic placental inflammation, a type of pathology linked to other neurologic morbidities (24, 25). Overall, there is a paucity of existing evidence directly linking placental disease to neonatal stroke.

The objective of this study was to determine the birth prevalence of perinatal stroke in term born infants at our high-volume delivery center, describe the clinical factors associated with perinatal stroke in our cohort, and assess the frequency of both gross and histologic placental pathologies associated with perinatal stroke using the Amsterdam Placental Workshop Group Consensus Statement guidelines and definitions. We hypothesized that perinatal stroke would be associated with acute inflammation of the placenta, maternal pregnancy complications, fetal congenital heart disease, and birth asphyxia.

## Subjects and methods

### Study subjects

This single-center retrospective cohort study was approved by the Institutional Review Board at University of Texas Southwestern Medical Center. All infants born at Parkland Hospital between 2010-2020 with a diagnosis of stroke were evaluated for inclusion in the study. Control placentas were collected as part of a separate prospective study, previously reported (26) with inclusion criteria of gestational age of at least 37 weeks, singleton pregnancy without diagnosis of fetal anomalies or fetal growth restriction, and no history of fetal asphyxia.

Demographic information was collected on both mother and infant including maternal age, gestational age at birth, infant sex, race, and ethnicity. Maternal pregnancy complications, fetal diagnoses, and birth characteristics were collected and percentiles for anthropometric data (27). Specific factors affecting risk for placental pathology were collected including maternal gravida status, maternal diabetes mellitus (DM; both gestational and pre-gestational), any form of maternal hypertension, and presence of placenta accreta spectrum disease.

### Placental pathology

In general, the approach to selection of placentas for pathologic examination include any maternal or fetal pregnancy complication, or any abnormality during labor or delivery including the need for advanced neonatal resuscitation. The standardized approach of our institution to placental pathology has been previously described (24). In summary, gross examination includes assessment of the umbilical cord, membranes, and placental disc. The trimmed placental weight is measured after removal of umbilical cord, fetal membranes, and non-adherent blood clots. Gross pathologies include placental size abnormalities [small for gestational age (SGA), <10^th^ percentile; and large for gestational age (LGA), >90^th^ percentile], umbilical cord anomalies, and aberrant placental disc shapes. To determine histopathology, placental discs are sectioned at 1-2 cm intervals and representative sections of parenchyma, as well as umbilical cord and fetal membranes, are examined. Lesions identified during sectioning are also examined. Based on the Amsterdam criteria (28), histopathologic abnormalities were divided into the following subcategories:

1. Acute histologic chorioamnionitis (AHC), further categorized as maternal inflammatory response (MIR) and fetal inflammatory response (FIR), also classified by stages and grades.
2. Villitis of unknown origin (VUE) including low grade, high grade, and villitis with avascular villi.
3. Maternal vascular malperfusion (MVM), which encompasses maternal vascular lesions (e.g. decidual arteriopathy, mural hypertrophy, incomplete transformation of spiral arteries, and decidual necrosis), infarcts, hemorrhage or hematoma, thrombi (involving >5% of parenchyma), villous changes (e.g. chorangiosis, syncytial knots, distal villous hypoplasia, and accelerated villous maturation), and placental hypoplasia.
4. Fetal vascular malperfusion (FVM) further divided into two patterns, a) segmental FVM, indicating thrombotic occlusion of chorionic or stem villous vessels, or stem vessel obliteration, and b) global FVM, characterized by partially obstructed umbilical blood flow accompanied by venous ectasia, intramural fibrin deposition in large vessels, and/or small foci of avascular or karyorrhectic villi.
5. Other inflammatory lesions such as chronic deciduitis with plasma cells, massive chronic intervillositis, perivillous fibrin, and histiocytic intervillositis; and, delayed villous maturation, and villous edema.
6. Abnormalities in placental size – either small for gestational age (<10^th^ percentile, SGA) or large for gestational age (>90^th^ percentile, LGA).

### Statistical analyses

Maternal and infant patient characteristics as well as placental pathologies were analyzed with descriptive statistics. Values are presented as mean ± standard deviation or median (interquartile range) where appropriate.

## Results

### Study subjects

Between 2010 and 2020, there were 129,759 live births at Parkland Hospital and a total of 19 infants born at ≥37 weeks gestational age diagnosed with stroke or presumed perinatal stroke during the study period, giving a perinatal stroke birth prevalence of 1 in 6,829 live births. In our cohort from this community safety-net hospital, 84% of our patient cohort was Hispanic. Of the 19 stroke patients, 53% were female, their median gestational age at birth was 39 6/7 (29, 30), and median Apgar scores were 8 (4, 8) and 9 (7, 9) at one and five minutes (**Table 1**). Seizure was the presenting symptom in 74% of the cohort. The average age at onset of symptoms was 1.5 days (2.5 hours, 2.8 days) for those diagnosed in the neonatal period.

**Table 1:** Maternal and infant patient characteristics.

Maternal Characteristics	Stroke n = 18	Control n = 40
Age	28 (23.5, 33.5)	28 (23, 33)
Gravity	2 (1, 3)	3 (2, 4)
Parity	1 (1, 2)	3 (1, 3)
Pregnancy Complication		
Diabetes Mellitus	2 (11)	0 (0)
Preeclampsia	3 (17)	0 (0)
Advanced Maternal Age	4 (22)	2 (5)
Prenatal Infection	6 (33)	0 (0)
Oligohydramnios	4 (22)	0 (0)
**Infant Characteristics**		
Female Sex	9 (50)	16 (40)
Gestational Age at Birth	39 4/7 (39, 40)	39 (39, 40)
Apgar Score (1 min)	8 (3, 8)	8 (8, 9)
Apgar Score (5 min)	9 (6, 9)	9 (9, 9)
Birthweight (g)	3417 ± 452	3498 ± 385
Birth Head Circumference (cm)	34.5 ± 0.8	35.0 ± 1.0
Time of Presenting Symptom (DOL)*	1.5 (0.1, 2.8)	NA

*excludes one case of presumed perinatal stroke. NA, not applicable.

### Characteristics of stroke and risk factors

In our patient cohort, 13 had AIS, four had hemorrhagic stroke, and two were diagnosed with CSVT (**Table 2**). Of the patients with AIS, six were left-sided, four were right-sided, and three were bilateral. AIS was multifocal in eight patients and most commonly occurred in the distribution of the MCA. Hemorrhagic stroke presented on the left side in three patients and on the right in one patient. In addition to parenchymal hemorrhage, there was associated IVH in two patients. CSVT affected the dorsal aspect of intracerebral veins and vein of Galen in one patient and the deep medullary veins in the other patient. Neither of these patients had concomitant IVH. One or more perinatal risk factor was present in nearly every patient with maternal infectious concerns the leading maternal risk factor (occurring in 6 maternal patients) (**Table 3**). Maternal preeclampsia was diagnosed in three patients and diabetes mellitus in two. Meconium-stained amniotic fluid was common in our cohort occurring in 12 patients. In addition, non-reassuring fetal heart tones (defined as Category II or III fetal heart rate tracings) occurred in seven patients and delivery room resuscitation was required in nine patients that had stroke. A total of five stroke patients were diagnosed with hypoxic-ischemic encephalopathy and six patients had congenital heart disease, which was primarily ASD with or without VSD. Clinical chorioamnionitis was diagnosed in four patients, but histologic evidence of acute inflammation was more common, as described with placental findings.

**Table 2:** Detailed clinical information .

Patient	Sex	Race/Ethnicity	Gestational Age	Delivery	Apgar Scores	Clinical Presentation	MRI Findings	Placental Exam
1	M	Hispanic	term	C/S	4, 7, 8	encephalopathy after delivery	Hemorrhagic transformation of ischemic infarct – left temporal lobe, occipital lobe, parietal lobe; bilateral grade 1 IVH	Yes
2	F	Hispanic	term	C/S	3, 8	seizure @ 1 HOL	Multifocal bilateral ischemic infarcts – left parietal, occipital, frontal lobes, left posterolateral thalamus, right parietal lobe; MR angiogram with asymmetrically small right internal carotid artery with hypoplastic right A1 segment of the ACA	Yes
3	M	Hispanic	post dates	C/S	8, 9	seizure @ 2 DOL	Multifocal unilateral ischemic infarcts – left parietal lobe, left occipital lobe	No
4	F	Hispanic	term	C/S	9, 9	seizure @ 15 DOL	Multifocal unilateral ischemic infarcts – right precentral gyrus, lower centrum semiovale-corona radiata extending to caudate nucleus, posterior limb of internal capsule	No
5	F	Asian	term	SVD	6, 9	seizure @ 2 DOL	Multifocal unilateral ischemic infarcts – right frontal lobe, parieto-occipital region, posterior temporal lobe; hemorrhagic transformation	No
6	M	Hispanic	term	C/S	8, 9	seizure @ 5 DOL	CSVT (dorsal aspects of intracerebral veins and vein of Galen) with associated multifocal infarcts	Yes
7	M	Hispanic	term	C/S	9, 9	seizure @ 4 DOL	Multifocal unilateral ischemic infarcts – right frontal lobe, deep white matter in centrum semiovale and corona radiata	Yes
8	M	Hispanic	term	C/S	8, 9	hemiparesis @ <1 year	Remote ischemic infarct – left frontal and parietal lobes; MR angiogram with extremely diminished caliber of left internal carotid artery and left MCA	No
9	F	Black	term	C/S	1, 2, 3	seizure @ 1 DOL	Focal unilateral ischemic infarct – right frontal and parietal lobes	Yes
10	F	Hispanic	term	C/S	8, 9	seizure @ 2 DOL	Multifocal unilateral ischemic infarcts – left parieto-occipital lobe and posterior limb of the internal capsule	No
11	F	Hispanic	term	SVD	9, 9	seizure @ 1 DOL	Hemorrhagic transformation of ischemic infarct – right temporal and occipital lobes with midline shift and left lateral ventricular dilatation; MR venogram with severe compression of patent right transverse and proximal sigmoid sinuses secondary to hemorrhage	No
12	F	Hispanic	term	C/S	6, 8	apnea @ 4 HOL	Focal unilateral ischemic infarct – left internal capsule; MR angiogram with diminished flow of the left carotid terminus, A1 segment of ACA, and MCA secondary to thrombus	Yes
13	M	Hispanic	term	SVD	8, 9	seizure @ 2 DOL	Multifocal bilateral ischemic infarcts – parieto-occipital lobes, right greater than left associated	No
14	M	Hispanic	term	C/S	9, 9	seizure @ 3 DOL	Focal unilateral ischemic infarct – left frontoparietal lobe; MR arteriogram with occlusion at small branch of distal left MCA at level of the superior insula	No
15	F	Hispanic	term	C/S	2, 8	seizure @ 2 DOL	Focal unilateral ischemic infarct – left posterior insular cortex	Yes
16	M	Hispanic	term	C/S	8, 9	encephalopathy @ 2 HOL	CSVT of deep medullary veins with no associated hematoma or IVH	Yes
17	F	Hispanic	term	C/S	1, 6, 6	encephalopathy after delivery	Hemorrhagic transformation of ischemic infarct – left temporal lobe, periatrial white matter, putamen, and scattered areas of cerebellar parenchyma; grade II IVH	Yes
18	M	Hispanic	term	C/S	2, 7	seizure @ 9 DOL	Hemorrhagic transformation of ischemic infarct – left temporal and frontal lobes	Yes

**Table 3:** Stroke risk factors.

Patient	Perinatal Stroke Risk Factors			Other Clinical Findings
	Maternal	Peripartum	Neonatal	
1	severe preeclampsia	meconium, required DR resuscitation with respiratory assistance	moderate HIE with therapeutic hypothermia, VSD	none
2	none	prolonged rupture of membranes, non-reassuring fetal heart tones, meconium, required DR resuscitation with endotracheal intubation	none	none
3	hidradenitis suppurativa, oligohydramnios	meconium, chorioamnionitis	ASD	hypoglycemia, thrombocytopenia
4	none	none	none	none
5	none	chorioamnionitis, meconium, required DR resuscitation with respiratory assistance including intubation	none	none
6	none	non-reassuring fetal heart tones, chorioamnionitis, meconium, forceps	none	none
7	advanced maternal age	Non-reassuring fetal heart tones, prolonged rupture of membranes	none	hyperbilirubinemia
8	morbid obesity, recurrent dacrocystitis	oligohydramnios, meconium	none	none
9	severe preeclampsia	non-reassuring fetal heart tones, face presentation with difficult C/S extraction, required advanced DR resuscitation with chest compressions, epinephrine, and endotracheal intubation	moderate HIE with therapeutic hypothermia	anemia, hyperbilirubinemia, sickle cell disease
10	none	none	PFO, small muscular VSD	none
11	gestational hypertension, teen pregnancy	large for gestational age	PFO versus small secundum ASD, small muscular VSD	none
12	type 2 diabetes	meconium	PFO versus small secundum ASD, anemia	metopic synostosis with mild trigonocephaly and hypotelorism, congenital fusion of radius and ulna, global hypotonia, concern for Saethre-Chotzen syndrome
13	advanced maternal age, severe preeclampsia, varicella infection during pregnancy	none	hypoglycemia with negative metabolic workup	none
14	gestational diabetes, positive red blood cell antibody screening	none	early brief hypoglycemia	mild laryngomalacia
15	history of multiple ectopic pregnancies	chorioamnionitis, non-reassuring fetal heart tones, meconium, required DR resuscitation with respiratory assistance	small secundum ASD	none
16	trichomoniasis infection during pregnancy	non-reassuring fetal heart tones, meconium, required DR resuscitation with respiratory assistance	herpes simplex infection of mucous membranes, moderate HIE and underwent therapeutic hypothermia	none
17	advanced maternal age, gestational diabetes, positive Zika screen (IgG positive, IgM negative), asymptomatic bacteriuria	meconium, non-reassuraing fetal heart tones, nuchal cord x4, required DR resuscitation with respiratory assistance including intubation	moderate HIE and underwent therapeutic hypothermia, feto-maternal hemorrhage, thrombocytopenia at birth, initial hypoglycemia	non-occlusive IVC thrombus, postnatal transaminitis
18	grand multiparity (gravida 7), asymptomatic bacteriuria, oligohydramnios, gestational thrombocytopenia	meconium, required DR resuscitation with respiratory assistance	mild HIE, moderate secundum ASD	cholestasis, hypospadias, dysmorphic features with normal chromosomal microarray

### Gross and histologic placental pathology

Ten of our patients with stroke had placental pathologic examination performed (53%), and results are summarized in **Table 4**. Only one of these placentas had no gross or histologic abnormalities compared to 29 (72.5%) of Control placentas with no abnormalities. Three placentas in our Stroke cohort were found to be SGA and one was LGA while no size abnormalities were present in the Control group. One placenta from a neonate with stroke had borderline hypercoiled umbilical cord and none of the placentas had abnormalities of disc shape. Similarly, no cord insertion abnormalities were reported in either group. All but one placenta in the Stroke group had gross and/or histologic evidence of meconium staining while only four placentas in the Control group had evidence of meconium. Acute inflammatory lesions were highly prevalent in the Stroke cohort with five placentas diagnosed with acute histologic chorioamnionitis, all with Maternal Inflammatory Response (MIR) Stage 2, and nearly all Grade 1. Fetal Inflammatory Response (FIR) was present in six placentas: isolated FIR Stage 2, Grade 1 without MIR in one placenta; Stage 2, Grade 1 in four placentas; Stage 1, Grade 1 in one placenta; and Stage 3, Grade 2 in one placenta. Only one placenta from the Stroke group with MIR did not demonstrate associated FIR. Acute inflammation was the only lesions in the Control placentas, and MIR was present in 11 (28%) with six of those showing associated FIR. In the Stroke group, chronic inflammation in the form of VUE was described in three of the acutely inflamed placentas and was high-grade in each of those. No placentas had VUE with avascular villitis. MVM was described in four placentas from neonates with stroke, with three showing placental hypoplasia and one with extensive thrombosis, infarcts comprising >5% of the parenchyma, and diffuse fibrin. FVM was found in none of the study placentas. Chronic deciduitis with plasma cells occurred in two placentas from the Stroke group. Delayed villous maturation and villous edema were not present in any placentas.

**Table 4:** Placental gross and histopathologic findings.

Patient	Stroke Type	Placental Pathology				
		Summary Placental Findings	Acute Inflammation	Chronic Inflammation	Maternal/Fetal Malperfusion	Other Lesions
2	Ischemic stroke	1. One vessel umbilical vasculitis 2. Infarct (<5% of parenchyma) 3. Meconium exposure	X			
7	Ischemic stroke	1. Acute chorioamnionitis 2. Intervillous thrombus 3. Meconium exposure	X		X	
9	Ischemic stroke	1. Infarct (<2% of parenchyma) 2. Meconium exposure				
12	Ischemic stroke	No gross or histologic pathology				
15	Ischemic stroke	1. Severe acute chorioamnionitis 2. Three vessel umbilical vasculitis 3. Funisitis 4. Chorionic plate vasculitis 5. Meconium exposure 6. Borderline hypercoiled umbilical cord	X			X
1	Hemorrhagic Stroke	1. Acute chorioamnionitis 2. Single vessel umbilical vasculitis 3. Patchy chronic villitis 4. Focal basal villitis 5. Small for gestational age 6. Meconium exposure	X	X	X	X
17	Hemorrhagic stroke	1. One vessel umbilical vasculitis 2. Funisitis 3. Chorionic plate vasculitis 4. Meconium exposure	X			
18	Hemorrhagic stroke	1. Acute chorioamnionitis (stage 1) 2. Patchy (high grade) chronic villitis 3. Diffuse basal villitis 4. Small for gestational age 5. Meconium exposure 6. Chronic deciduitis with plasma cells	X	X	X	X
6	CSVT	1. Multifocal basal chronic villitis 2. Small for gestational age 3. Meconium exposure 4. Chronic deciduitis with plasma cells		X	X	X
16	CSVT	1. Slight acute chorioamnionitis 2. Single vessel umbilical vasculitis 3. Extensive intervillous thrombi 4. Infarcts (>5% of parenchyma) 5. Increased perivillous fibrin 6. Large for gestational age 7. Meconium exposure	X		X	X

## Discussion

In this single-center cohort study we demonstrated that 1) pathologic placental lesions often accompany perinatal stroke, particularly acute and chronic inflammatory conditions, and 2) placental examination is frequently missed in the clinical investigation to determine stroke etiology, even in centers with robust guidelines for placental pathology practices. Nevertheless, placental examination in 53% of our perinatal stroke cohort is significantly higher than prior studies where rates of placental examination for neonates diagnosed with stroke ranged from 6-13% (31). The most likely explanation for the low rate of placental pathology in these cases is the delayed onset of symptoms and diagnosis of perinatal stroke, by which time the placenta may have already been discarded.

Perinatal stroke is an infrequent but important cause of both immediate and long-term morbidity in neonates. Our single-center data show an overall birth prevalence of perinatal stroke of 1 in 6,829 over a 10-year period at our high-volume delivery center, which differs from other reports and indicates that some patients may have gone undiagnosed during postnatal hospital stay. Furthermore, our report includes only one case of presumed perinatal stroke, which likely contributes to the difference we report in birth prevalence of perinatal stroke compared to other studies (1, 18, 32, 33). Since our NICU readmits patients from home for a limited range of indications only within the first week after birth, these results exclude many patients who are later diagnosed due to seizures, early handedness, hemiparesis, or delayed achievement of developmental milestones. This brings attention to the fact that perinatal stroke diagnosis requires a high level of clinical suspicion and for many patients, may go undetected until later. Presumed perinatal stroke accounts for a significant number of perinatal stroke cases and typically presents before one-year of age (3, 34).

While numerous studies have outlined risk factors associated with perinatal stroke (12, 15, 21, 34, 35), the exact etiology remains unknown and is likely multifactorial, including maternal, peripartum, and neonatal factors. Nearly all our patients had one or more perinatal risk factor(s) and seizures were the most frequent presenting symptom in our cohort, as has been previously reported (5). In a meta-analysis of eight studies including 550 cases of neonatal AIS, maternal preeclampsia imparted a significantly increased risk of neonatal AIS (36). In our small cohort, preeclampsia was less prevalent than maternal infectious concerns which ranged from asymptomatic bacteriuria to hidradenitis suppurativa. In addition, clinical chorioamnionitis was diagnosed in 60% of the maternal patients in our cohort. In prior reports, inflammatory conditions including chorioamnionitis have been prominent risk factors for perinatal stroke (31).

Our understanding of the role of the placenta in perinatal AIS has evolved in recent years with new evidence emerging that there are high rates of multiple placental histopathologies in perinatal stroke, particularly AIS (37, 38). Our cohort confirms those findings with 90% of stroke patients having at least one placental abnormality. This is similarly high to the rate of placental lesions in a cohort of patients we recently studied with congenital heart disease (78%) (39). Placental inflammatory conditions seem to play a prominent role in perinatal stroke pathophysiology (38), and our cohort has similar findings with 80% showing acute and/or chronic inflammation of the placenta. These inflammatory processes may contribute to formation of emboli of placental origin that reach the fetal cerebrovasculature through the PFO (38, 40). Further evidence supporting a placental origin of emboli are that an estimated 30% of neonatal stroke is multifocal, even in the absence of CHD and the recurrence of AIS postnatally is exceedingly rare (4). Infarcts were multifocal in 42% of our cohort and to our knowledge, none of the cohort experienced stroke recurrence, although our study is limited by the information available in the medical records. Our study was also limited by the small number in our cohort, the potential for ascertainment bias in the placentas sent for pathologic examination, and the limited outcome data available. Similarly, nearly half of the cohort did not have placental pathologic examination performed, which underscores the selection bias involved in placentas sent for pathology, although our center has a culture of commonly sending placentas to pathology for any indication related to maternal or fetal pregnancy or peripartum risk factors such as pregnancy complications (e.g. maternal diabetes mellitus, fetal growth restriction), or abnormalities during labor or delivery (e.g. perinatal asphyxia, placental abruption).

The suspected pathophysiology of AIS supports the clinical practice of placental pathologic examination in all cases of perinatal stroke (1, 29, 30); however, this retrospective approach is inadequate to change clinical outcomes or prevalence of perinatal stroke. New technologies are required to change perinatal stroke care from a focus on diagnosis and recovery, to one of prevention. This will require creative utilization of what we already know as well as development of new technologies. Recent studies have focused on identifying inflammatory biomarker signatures in patients at-risk for perinatal stroke (41), which may improve the ability to detect perinatal stroke early and allow initiation of supportive therapies sooner. Some experts have recommended more widespread use of neuroimaging in neonates with risk factors for stroke (42), but clear algorithms are still lacking to define which neonates are at highest risk. Placental pathology may provide input into a stroke risk stratification algorithm, but the timeframe required for placental examination will be a limiting factor until new techniques are developed that can offer real-time, clinically-relevant information. In order to establish preventative strategies, advanced methods of *in utero* assessment are needed, such as the many emerging methods in advanced placental magnetic resonance imaging (43–46).

## Conclusion

Inflammatory conditions including maternal infection and fetal asphyxia are often present in patients with perinatal stroke. Placental pathologic examination has a high yield of abnormal findings and should be considered integral to the diagnostic workup in all neonates with perinatal stroke. Imaging or blood biomarkers may eventually play a role in assessing those at risk for perinatal stroke in order to employ timely and effective interventions.

## Data availability statement

The original contributions presented in the study are included in the article/Supplementary Material. Further inquiries can be directed to the corresponding author.

## Ethics statement

The studies involving human participants were reviewed and approved by University of Texas Southwestern Medical Center Institutional Review Board. Written informed consent from the participants’ legal guardian/next of kin was not required to participate in this study in accordance with the national legislation and the institutional requirements.

## Author contributions

Concept: LC, IM, RL. Data Curation: LC, IM. Investigation and Analysis: RL, VK, MA, JT. Drafting, Revising: RL, VK, MA, JT, IM, LC. Final Approval: RL, VK, MA, JT, IM, LC.

## Conflict of interest

The authors declare that the research was conducted in the absence of any commercial or financial relationships that could be construed as a potential conflict of interest.

## Publisher’s note

All claims expressed in this article are solely those of the authors and do not necessarily represent those of their affiliated organizations, or those of the publisher, the editors and the reviewers. Any product that may be evaluated in this article, or claim that may be made by its manufacturer, is not guaranteed or endorsed by the publisher.
